# Confronting the Business Models of Modern Slavery

**DOI:** 10.1177/1056492621994904

**Published:** 2021-02-25

**Authors:** Andrew Crane, Genevieve LeBaron, Kam Phung, Laya Behbahani, Jean Allain

**Affiliations:** 1 University of Bath, Bath, UK; 2 University of Sheffield, Sheffield, UK; 3 York University, Toronto, ON, Canada; 4 Simon Fraser University, Burnaby, BC, Canada; 5 Monash University, Clayton, VIC, Australia

**Keywords:** business models, business model innovation, modern slavery, forced labor

## Abstract

Despite growing attention from companies and regulators looking to eradicate modern slavery, we know little about how slavery works from a business perspective. We address this gap by empirically examining innovations in the business models of modern slavery, focusing on how the business models of slavery in advanced economies have evolved since slavery was legally abolished. While continuities exist, novel business models have emerged based on new actors, activities, and linkages. We categorize these as four innovative models per actors involved (producer/intermediary) and how value is created and captured (revenue generation/cost reduction), and discuss implications for research, policy, and practice.

Modern slavery is one of the most extreme forms of labor abuse in the global economy. Defined as “situations of exploitation that a person cannot refuse or leave because of threats, violence, coercion, deception, and/or abuse of power,” tens of millions of people are estimated to be in modern slavery every year (International Labour Office & Walk Free Foundation, 2017). As such, it has become one of the defining grand challenges of our time. In a business context, modern slavery usually manifests as human trafficking, debt bondage, and forced labor, all of which have been legally prohibited in most countries in the world for decades—and in many cases for nearly two centuries—in light of the considerable suffering and unfreedom inherent in these forms of labour exploitation.^1^

Despite such proscriptions, modern slavery flourishes. Take the example of the Thai fishing industry, where for years large numbers of men have been forced under threat of violence to work for no pay in the production of seafood destined for retailers such as Costco, Walmart, Tesco, and Carrefour (Hodal et al., 2014; Lawrence & Hodal, 2017). Or consider the Malaysian electronics industry, where forced labor has been revealed to be “systematic,” with more than a third of migrant workers experiencing debt bondage and the confiscation of their passports to prevent them from leaving their employment (Kelly, 2014). Indeed, forced labor has been identified in an array of industries and countries ranging from coltan mining in the Democratic Republic of the Congo to cocoa farming in Côte d’Ivoire to construction in Qatar (Verité, 2017). Irrespective of widespread agreement that slavery should have no place in our society—as demonstrated by various national and international laws as well as transnational agreements including the UN Declaration of Human Rights and the 1930 ILO Forced Labor Convention—it seems to be present almost everywhere.

The prevalence of such extreme forms of exploitation has prompted an emerging stream of research on the business of modern slavery, covering such issues as the role of supply chain management practices in contributing to modern slavery, the social responsibility strategies that firms might deploy to tackle modern slavery, the role of consumers in influencing company responses, and the effectiveness of regulation in changing corporate behaviour (Caruana et al., 2021). These studies primarily focus on the role of big brands in taking responsibility for and addressing modern slavery in their supply chains, particularly in the context of anti-slavery regulation requiring greater supply chain transparency among firms, including the California Transparency in Supply Chains Act, 2010 and the UK Modern Slavery Act, 2015 (e.g., Birkey et al., 2018; LeBaron & Rühmkorf, 2017; Stevenson & Cole, 2018).

In contrast, there has been very limited attention paid to the firms in those supply chains actually engaged in modern slavery—that is, organizations directly involved in perpetrating slavery (Phung, 2018). Although some research has made important first steps in indentifying the competences required by such firms to exploit institutional conditions giving rise to modern slavery (Crane, 2013), “we know remarkably little about the business and organisational dynamics of forced labor, including how and why it is deployed as part of a business model” (LeBaron & Crane, 2019, p. 26). Indeed, there has been scant research on the creation of business models to practice modern slavery beyond rather superficial, and widely critiqued, accounts in the popular press.^2^ However, confronting these business models is important because if companies, governments, and nongovernment organizations are seeking to identify, reduce, and eradicate modern slavery, they will need to understand how it operates in terms of its business models to develop effective interventions. Combatting modern slavery requires a better knowledge of how and why it flourishes in business and how such knowledge can be leveraged to impede the operation of enterprises using such practices in the context of the broader political economy dynamics that give rise to severe labor exploitation in the global capitalist economy (LeBaron, 2020).

Yet, our ability to combat modern slavery will be limited without a robust understanding of how businesses deploying slavery in contemporary society have evolved over time, and especially, how business models have evolved since slavery was widely abolished and rendered illegal. In contrast to modern slavery, the business of traditional slavery has been extensively researched (e.g., Ransom & Sutch, 1977; Ruef, 2014; Schermerhorn, 2015; Williams, 1944). While much has been written about the differences and similarities between traditional and modern forms of slavery (e.g., Bales, 2004; Quirk, 2006), this scholarship has yet to specifically address business models. The business models of modern slavery, though, might be expected to deviate quite substantially from those used in traditional slavery. Not only has business itself been transformed since that time, but slavery is also now an illegal practice that can cause reputational damage and negative attention for business. While the UN has estimated that modern slavery is “a $150bn-a-year business” (International Labour Office, 2014), it is unclear what type of business it is, how it generates such profitability, and how—from a business point of view—it has evolved from more traditional forms of slavery. Assumptions that the strategies underlying modern slavery are either similar or different to those of traditional slavery are not helpful for designing interventions unless based on sound research and analysis. Accordingly, in this study we ask the following: *How can we understand the contemporary business models of modern slavery, and what novel distinctions have emerged in relation to the business models of traditional slavery that need to be understood and disrupted?*

We explore this question through an inductive case study of modern slavery across three sectors (cannabis, construction, and food) in the United Kingdom, focusing on what we term “modern slavery enterprises”—that is, organizations, or in some cases even individuals, that are directly involved in perpetrating modern slavery, which are typically small or medium sized, and are often informal or criminal enterprises, rather than mainstream companies. We focus on modern slavery enterprises in the United Kingdom engaged in modern slavery practices within the country. We ground our comparative analysis in the business models that prevailed in the 19th-century Antebellum US South (Johnson, 2013, Miller & Smith, 1997; Rosenthal, 2018), since these are widely viewed as emblematic of traditional slavery. Although UK companies also used slavery in the 19th century (Smith & Johns 2019) and would also make for a sound reference point, we opted to ground our comparison in the business models of slavery plantations physically located in the United States (irrespective of the nationality of their owners) since—as we discuss further—this context (the 19th-century Antebellum US South) is commonly invoked and used as a basis for comparison between “traditional” and “modern” slavery within contemporary social science scholarship (Azmy 2002; Bales, 2004), and is frequently (if not accurately) depicted as the “typical” instantiation of slavery (Pargas, 2016).

Our findings reveal that the business models of modern slavery can be best understood in terms of two dimensions: whether the slavery enterprise is a producer or intermediary, and whether it captures value through revenue-generation or cost-reduction activities. Moreover, our dissection of business models along these dimensions reveals four business models of modern slavery that are distinctly novel in comparison to the business of traditional slavery: risk reduction, asset leveraging, evading legal minimums, and workers as consumers. Ultimately, we show that while continuities from the business models of traditional slavery persist in contemporary society, key divergences in the business models of slavery have emerged as part of the evolution of slavery into present times.

In identifying and dissecting the novel business models of modern slavery, we confront and and bring clarity to the vagueness surrounding the businesses often situated at the bottom of supply chains and in the shadows of the economy that actually perpetrate modern slavery, thereby addressing a frequently neglected part of the modern slavery puzzle and literature (e.g., LeBaron & Crane, 2019; Phung & Crane, 2018). In addition, by framing our analysis of these changes in terms of business model innovation (e.g., Amit & Zott, 2012; Osterwalder & Pigneur, 2010; Teece, 2010), we contribute a novel dark side analysis to this literature, offering stark examples of and pathways for negative business model innovations anchored in oppression and exclusion (Martí, 2018). Finally, we reveal how understanding the novel distinctions of the business models of modern slavery can inform policy makers and practitioners in their efforts to design interventions to combat modern slavery, recognizing the essential role of governments in either facilitating or helping to eradicate modern slavery and in developing effective ways to rid supply chains of labor exploitation (e.g., Crane et al., 2019; LeBaron, 2020).

The remainder of this paper proceeds as follows. First, we explore the business of traditional slavery and locate these practices within a theoretical framing of business models. Next, we specify our research questions and methods, detailing our empirical study of modern slavery in the United Kingdom. Then, in our findings, we present a typology of novel business models before finally discussing the implications of our analysis for theory, practice, and the design of effective interventions.

## The Business of Traditional Slavery

Today, slavery is considered an “obvious wrong” (Quirk, 2008), but insofar as this is true now, it has not always been the case. Traditional slavery (i.e., that which took place prior to its legal abolition across the 19th century) covered many forms and contexts but was largely accepted (or at least tolerated) as a necessary social and economic institution that was protected in law and custom. Historical research has documented variations in how such traditional slavery operated as a business across periods and regions (Black 2011; Quirk & LeBaron, 2015; Sinha, 2016). Without discounting the differences that characterized slavery prior to its abolition, we use slavery in the Antebellum South as a key reference point for comparison because (a) it has been subjected to the most comprehensive economic analysis to date with respect to the business of slavery; (b) it is the most common reference point within the literature on modern slavery (e.g. Bales, 2004); and (c) it represents a similar basis for comparison for our empirical material that relates to an advanced capitalist country, particularly given that national setting is far less relevant to our interest in this paper than business type and dynamics. Indeed, while our contemporary empirical focus is on modern slavery that takes place in the United Kingdom and traditional slavery that took place in the Antebellum US South, historians have shown that UK companies were also heavily involved in slavery in the United States during this period (both before and after independence) and that US slavery was very much a joint Anglo-American effort supported by the British empire (Beckert, 2015; Boodry, 2016).

While there has been much debate about the economic rationality or otherwise of slavery (Adam Smith, 1981, for example, argued that slavery was economically inefficient and was motivated by “love of domination”), it is by now widely accepted that despite what we might now think about the ethical unacceptability of slavery, plantation owners in the Antebellum South, “like profit-maximizing businessmen.. chose the most profitable investments available” (Miller & Smith, 1997, p. 595). According to Stampp (1956, p. 85), “slavery in the South was the most productive and profitable mode of production of this era” as slaves worked year-round and compensated for price declines. Planters could extract more labor hours per worker per year than in the North (Anderson & Gallman, 1977; Wilson, 1996). Defining efficiency as the ratio of output to the average input, Fogel and Engerman (1974, p. 192) found that “southern slave farms were 28 percent more efficient that southern free farms. . .[and] compared with northern farms. . .slave farms were 40 percent more efficient.” This is not to suggest that the labor of enslaved people was a mechanical input; indeed, as historians like Walter Johnson have extensively argued, “while it is easy to lose sight of the elementally human character of labor—even that of forced labor—in light of the salutary political effect of labelling slavery ‘inhuman’,” the enslaved exhibited agency within these business models, defining and redefining labor processes, even as they were subjected to violent physical punishment, coercion, and pain (Johnson, 2013, p. 9).

Thus, scholars have shown that planters used slavery because, notwithstanding any moral considerations, they recognized that certain crops could be profitable when produced to scale and that slave labor was reliable, allowed for more control, and was more productive than free labor (Clark, 2012; Klein, 2007; Morgan, 1975; Williams, 1944). As Williams (1944, p. 19) notes, “the cheapness of the labor” made slavery the economically superior option. For example, Wilson (1996, p.71) shows that a planter who invested $300,000 for 300 slaves, “earns back this initial investment in 4 years and continues to accumulate more than $79,000 annually in labor cost savings.” Having established, albeit quite briefly, that antebellum US slavery was motivated by profit and that antebellum plantations functioned as profit-maximizing firms, we now turn to its underlying business model.

### Business Models and their Application to Traditional Slavery

The business model is central to contemporary thinking about commercial enterprises, and it has spawned a vast body of academic literature that represents an important extension to the traditional strategy literature in management (Massa et al., 2017). While the business model continues to suffer from a lack of definitional and conceptual clarity (Demil et al., 2015; Zott et al., 2011), “[a]t a very general and intuitive level, *a business model is a description of an organization and how that organization functions in achieving its goals* (*e.g., profitability, growth, social impact*)” (Massa et al., 2017, p. 73, italics in original). The business model is also commonly regarded as a unit of analysis, a system-level concept centered on activities and focusing on value (Zott et al., 2011), and it has been increasingly applied to the study of environmental and social issues, including oppression (Martí, 2018), as well as how businesses respond to a changing societal landscape.

We view a business model as “the rationale of how an organization creates, delivers, and captures value” (Osterwalder & Pigneur, 2010, p.14). In turn, it concerns the specification of the organizational resources, revenue streams, and cost structures that give rise to profitability. Put simply, “a good business model answers. . .the fundamental questions every manager must ask: How do we make money in this business? What is the underlying economic logic that explains how we can deliver value to customers at an appropriate cost?” (Magretta, 2002, p.4).

The way that researchers of business models conceptualize transitions and evolutions in business models is through the lens of “business model innovation,” which by extension to the definition of a business model represents a new way of aligning resources, revenues, costs, and value to make money. According to Amit and Zott (2012), business model innovation can involve adding new activities, linking activities in new ways, or changing which actor performs an activity. Such innovations can create new markets, exploit new opportunities, reshape industries, generate and capture new value, create barriers to entry, adapt to changes, and offer competitive advantages, but they do not come easily and they often require trial and error, experimentation, and patience (Amit & Zott, 2012; Chesbrough, 2007, 2010; Johnson et al., 2008; Osterwalder & Pigneur, 2010; Teece, 2010). To date, business model innovation has been almost entirely framed as a positive development that enables companies to create value in new ways. However, in using the concept in the context of slavery, we want to highlight that evolutions of business models are far from always positive and that they can also have profoundly negative societal effects and entail considerable harm and abuse for workers.

Returning to slavery in the Antebellum South, we can make a case, distasteful as it might be, that Southern plantations that used slave labor relied on specific business models and that these business models were in some respects an “innovation” on the business models of plantations that used free labor. Of course, this business “innovation” involved far more severe forms of labor discipline and the imposition of greater levels of unfreedom than were generally present among businesses who relied on so-called free waged labor and was therefore more just an increase in brutality and terror from labor’s point of view. Likewise, we will argue that business models of modern slavery are distinctively novel when compared to antebellum slavery, representing highly negative transformations that enable businesses to continue to profit from slavery despite its illegality. In this, we are following Martí (2018) in examining the dark side of business model innovation, where rather than only being concerned with social or economic progress, we can also examine how new business models can contribute to oppression and exclusion.

According to Amit and Zott’s model (2012), we suggest that slavery plantations changed their business models by changing *who* performed key value creating activities since they relied on enslaved labor rather than waged labor. Enslaved labor created an advantageous cost structure, converting a variable cost to an upfront fixed cost with a modest payback (Anderson & Gallman, 1977; Metzer, 1975). Planters could extract more work from enslaved laborers, which enabled them to produce to scale, diversify their crops, internalize services, and become self-sufficient. Thus, they minimized their costs, improved profits, and often generated more revenue. Plantations using enslaved labor also created relationships with key partners such as slave traders, and they continued to persist because it was supported by government policy. Therefore, changes in one area (i.e., who performed activities) enabled other business model changes. For instance, it facilitated vertical integration to internalize services that would have been contracted out to free labor. While a full analysis of the business models of traditional slavery is beyond our scope, it is clear that slavery changed the business model of the Southern plantation in ways that facilitated business profit as well as high levels of human suffering.

Despite the economic advantages of the slavery business model, it eventually was dismantled by a decades-long anti-slavery movement, which included successful attempts to undermine the business model of slavery, including consumer boycotts in the United Kingdom that targeted slave made imports (Smith & Johns, 2019). As Quirk (2006, p. 584) argues, this was a “remarkable achievement. . .against an enduring, valuable and historically entrenched institution. The outcome of this confrontation was by no means inevitable. The obstacles involved were substantial, the costs entailed considerable, yet slavery was gradually stripped of legal standing.” The arguments against slavery, which seem so taken for granted now, revolved around the injustices of ownership of other human beings, and extreme exploitation (Quirk 2006). However, following the legal abolition of slavery, which was enacted in the United Kingdom in 1833 and in the United States in 1865, these problems did not cease entirely but continued in a variety of forms and designations (Quirk, 2008), including in mining (Evans, 2013) and cocoa farming (Satre, 2005). Today, the term “modern slavery” is used to refer to some of the most extreme forms of exploitation that have some degree of equivalence to traditional slavery, albeit without the legal status that slavery once enjoyed. Thus, our guiding research question is: how can we now understand the business models of modern slavery in relation to those of traditional slavery, and what novel distinctions have emerged that need to be understood and disrupted?

## Methods

### Research Context and Design

To explore this question, we conducted a qualitative case study of modern slavery in contemporary United Kingdom. While a crucial step in qualitative research is finding a “theory-method fit” (Gehman et al., 2018), “theory building from case study research is particularly appropriate” when “little is known about a phenomenon, current perspectives seem inadequate because they have little empirical substantiation, or they conflict with each other or common sense” (Eisenhardt 1989, p. 548). For modern slavery, “not only does the field lack a deep theoretical understanding on modern slavery, but it also suffers from deficiencies in terms of its empirical understanding at the organizational level and of the overall business side” (Phung & Crane, 2018, p. 180).

Modern slavery is an umbrella term that covers a wide range of practices, including forced labor, forced marriage, domestic slavery, child soldiers, forced prostitution, and human trafficking, and it occurs in a variety of contexts. For empirical precision, we focused specifically on forced labor imposed by private actors for the purposes of labor exploitation since this was most relevant to our research question. We also focused on the context of the United Kingdom because selecting a developed country that had abolished and criminalized the practice of slavery enabled us to focus on specifically modern forms. Further, the fact that the United Kingdom has been at the forefront of government efforts to combat modern slavery makes it an ideal context in which to explore how businesses have managed to evade laws banning the use of severe labor exploitation. We do not claim to be exhaustive in our study of modern slavery, and caution should be made when generalizing beyond the sectors and context that comprise the focus of our study.

In empirically operationalizing the umbrella concept of modern slavery in terms of the more specific practice of forced labor, we applied the internationally recognized definition of forced labor from the International Labour Organisation Forced Labour Convention.^3^ Thus, we considered someone as being in forced labor when their work was involuntary because of force, fraud or deception, and a penalty or threat of a penalty being used to coerce them (for a longer discussion of operationalizing the ILO definition of forced labor for social science research see LeBaron, 2018). We also concentrated on two legal business sectors (food and construction) and one illegal business sector (cannabis cultivation) to explore different business models across formal, informal, and criminal modern slavery enterprises.

### Data and Analysis

To explore the business side of modern slavery in our three focus sectors in the United Kingdom, we collected data from two main sources: (a) archival data and (b) interviews. We drew on archival data because forced labor in the United Kingdom had experienced notable attention in the public and scholarly domains, and we knew also that there was existing and detailed court documentation that we could draw on. Our archival data consisted of a total of 62 court documents and appeal cases, 35 newspaper articles, 42 academic studies, and 63 reports that offered detailed insight into well-documented cases in our three sectors.

We conducted 33 semi-structured interviews with key informants in the field (Table 1). Evidently, due to the sensitivity of the issue, collecting primary data on perpetrators of modern slavery presents grave challenges (Stringer & Simmons, 2015) and has hindered the ability of researchers to understand the business side of modern slavery (LeBaron & Crane, 2019). However, when collecting data directly from the source (i.e., perpetrators of modern slavery) is not an option, adopting a stakeholder-oriented approach with informants knowledgeable about such practices, but not directly involved in conducting them, can be an effective way to piece together the puzzle (Phung & Crane, 2018). Thus, we conducted interviews with experts on forced labor, law enforcement agents, governmental and non-governmental organization (NGO) representatives, researchers, journalists, lawyers, employer representatives, company executives, consultants, social auditors, and trade union representatives, all with knowledge of at least one of our sectors and often more. All interviews were audio-recorded and transcribed for analysis.

**Table 1.** Overview of Interviews.

Position	Industries Discussed
Director, NGO	Food, construction, cannabis
CEO, NGO	Cannabis, food, construction
Communications officer, Trade union	Food, construction
Director, Public body	Food
President, Corporation	Construction
Director, Trade association	Food
Executive, Social audit firm	Food, construction
Head of advocacy, NGO	Cannabis
Sustainability manager, Audit firm	Food, construction
Director, Consulting firm	Cannabis, food, construction
Journalist & consultant	Food, construction
Executive director, NGO	Food, construction, cannabis
Director, NGO	Cannabis
Senior manager, Law enforcement	Cannabis
Director, Multi-stakeholder initiative	Food
Regional Officer, Trade union	Food, construction
Drugs strategy manager, Law enforcement	Cannabis
Director, Social audit firm	Food, construction
Policy officer, Trade union	Construction
Drugs expert, Law enforcement	Cannabis
Chief executive officer, NGO	Cannabis, food
Associate professor, University	Cannabis
Sector manager, Risk management firm	Construction
Vice president, Non-profit organization	Food, construction
Criminal intelligence analyst, Law enforcement	Cannabis
Chief executive, Public body	Food
Manager, NGO	Food, construction
Manager, Recruitment company	Construction
Manager, NGO	Food, construction
CSR team, food retailer	Food
Manager, Recruitment company	Food
Professor, University	Food, construction, cannabis
Manager, Consulting firm	Food

We acknowledge that our approach limits, to some extent, our ability to give direct voice to the victims of modern slavery. Conducting on the ground research with workers themselves is important since no matter how exploited, workers always have agency and a perspective on their conditions and can be a valuable source of information about the business dynamics of modern slavery (LeBaron, 2018, 2020; LeBaron & Crane, 2019). However, this type of research is challenging and resource-intensive to conduct ethically and, although we have undertaken extensive research with workers in other settings and believe it is important to give voice to those confronting labour exploitation within research on the topic where it is possible to do this well, it was not feasible to integrate into our research design for this particular project. We have therefore sought to interview actors who have extensive direct contact with victims and to draw on sources that give voice to victims to shine light on their lived experiences. For example, the report by Elliot and Lucio (2011) in our database is “primarily a collation of individual stories that seeks to capture something of the day-to-day experience of vulnerable workers in construction,” while that of Kagan et al (2011) adopted a life-story interview method with migrant workers.

These kinds of “modern slave narratives” are important in making modern slavery visible and legible, giving voice to the variety of experiences of enslavement and exploitation, and articulating potential solutions from the point of view of those affected (Johnson, 2013; Murphy, 2015). Drawing from our data, we showcase vignettes and extended quotes from reports and other sources to give a stronger flavor of the victim experience of modern slavery business models. Nevertheless, these accounts remain framed and re-presented by us, as well as other researchers, in order to help tell our specific interpretation of their stories. As such, we have sought to avoid common problems in third-person representations of the experiences of modern slavery victims such as “mak[ing] a spectacle of the enslaved body” (Murphy 2015, p. 394) and reducing the variety of experience to a prototypical slavery “script” (Johnson, 2013).

Our analysis followed the commonly prescribed approach to coding data, in which we iteratively developed multiple levels of codes (Miles & Huberman, 1994). Specifically, our analysis involved three levels of coding: (a) initial coding, where empirical themes that emerged from the interviews were linked with those extracted from the secondary data in relation to the components of business models derived from the literature, such as “price of service”, “labour cost”, “profit”, etc; (b) categorical coding, where initial codes were refined and examined to determine specific manifestations of these components in a business model, such as “value creation”, “value capture”, “cost reduction”; “revenue generation”, etc; and (c) thematic coding, where themes were identified from the refined data and compared with the literature to develop our business models and their dimensions. We then convened a round-table discussion with eight experts in the field to refine and validate our interpretations. The process of analysis was iterative but not linear. That is, we moved constantly between data and interpretation, refining codes as we progressed, adjusting our theoretical framework as new insights emerged, and repeatedly returning to our data to investigate interesting avenues for further exploration. We also sought not to abstract our analysis of business models too far from the embodied experiences of enslavement by victims, so our analytical categories remained tethered to specific cases and vignettes throughout the analytical process. We continued in this way until we had developed a logically consistent and theoretically sound interpretation that was consistent with our data.

## Findings

While traditional slavery gave rise to a relatively simple and stable business model supported by institutions, modern slavery has encompassed a variety of business models with different degrees of complexity. We first outline a framework that parsimoniously captures the essential contours of the business models of modern slavery. Then, we discuss how these differ from those that characterize traditional forms of slavery.

### Confronting the Business Models of Modern Slavery

**Figure 1.**
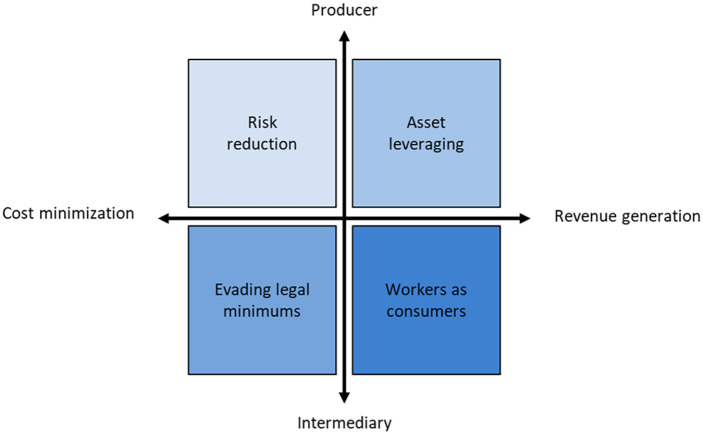
New business models of modern slavery.

Our analysis suggests that the business models of modern slavery can be best captured with respect to two key dimensions: (a) which actor is directly capturing value from modern slavery (i.e., the modern slavery enterprise), which we distinguish between producers and intermediaries; and (b) through which activities or combinations of activities do actors create or capture value, which we distinguish between revenue generation and cost reduction activities. Thus, while it entails abhorrent living and working conditions for labor, modern slavery can improve an enterprise’s profit formula through its cost structure, revenue stream, or both (Johnson et al., 2008; Osterwalder & Pigneur, 2010). Combining these dimensions, we identify and dissect four business models of modern slavery that possess key distinctions in comparison to traditional slavery: risk reduction, asset leveraging, evading legal minimums, and workers as consumers (see Figure 1).

Although we have discussed the two dimensions of the framework in Figure 1 in terms of two main forms (producer-intermediary and cost minimization-revenue generation), different business models and the employment relationships they describe are likely to exhibit different degrees along these dimensions. For instance, some employment relationships will have greater degrees of intermediation than others (e.g., when multiple intermediaries are involved). Similarly, some business models will exhibit a higher degree of revenue generation. As such, we have represented them as continua with axes pointing outwards and not discrete categories.

Each of these business models comes in different forms and they vary in terms of the nature of perpetrators, victims, value practices (i.e. practices deployed to create or capture economic value) that give rise to exploitation, and the focus of their modern slavery business model. While these variations are summarized in Table 2 and discussed in detail further, we also offer illustrative vignettes of victims of the identified modern slavery business models in Table 3 to illuminate the lived experiences of victims and humanize our study.

**Table 2.** Elements of Modern Slavery Business Models.

Modern Slavery Business Models		Perpetrator	Victim	Value Practices that Give Rise to Exploitation	Focus of Modern Slavery Business Model
**Risk reduction**	**Illegal labor practices**	Producer in legal industry, subject to external oversight due to geographic location or supply chain position.	Vulnerable workers, usually with poor skills, language, and understanding, plus mistrust of authorities.	Use of coercion to force workers to hide value capturing practices.	Reducing costs by reducing risks of illegality.
	**Illegal markets**	Producer in illegal industry, usually organized criminal gang.	Trafficked workers, usually of same national origin as perpetrator.	Use of coercion and physical restraints to force workers to hide value creating practices.	Reducing costs by reducing risks of illegality.
**Asset leveraging**	**Leveraging organization’s assets**	Producer in legal industry with low potential for value capture within the supply chain from production.	Vulnerable workers, especially those with little capital and/or in debt.	Sale of ancillary services at excessive prices to increase control and avoid value distribution to workers.	Generating additional revenue from activities related to core business to recoup labor costs.
	**Leveraging workers’ assets**	Producer in legal/illegal industry, sometimes informal business, with low potential for value capture from production.	Vulnerable workers, usually resident within the relevant local welfare regime.	Use of coercion to extract value from victim’s assets.	Generating additional revenue from activities unrelated to core business.
**Evading legal minimums**	**Subcontracting to other intermediaries**	Informal labor intermediary, usually same ethnicity as victims.	Vulnerable workers, often migrants recruited in home country.	Create value for producers and other intermediaries by offering low-cost labor provision that avoids statutory labor standards.	Reducing labor costs through informal labor contracting.
	**Exploiting regulatory loopholes**	Usually formal labor intermediary, possibly operating across borders.	Vulnerable workers, usually migrants, possibly recruited overseas.	Create value for producers and other intermediaries by offering low-cost labor provision that avoids statutory labor standards.	Reducing labor costs through regulatory avoidance.
**Workers as consumers**		Formal or informal labor intermediary, with low potential for value capture from core service.	Vulnerable workers, usually migrants.	Capture value from sale of ancillary services at high prices to maximize revenue per worker.	Generating revenue by exploiting control over indebted workers.

**Table 3.** Vignettes of Victims of Modern Slavery Business Models.

**Risk reduction**
Vignette from the cannabis industry (Kelley, 2019) Minh was a 16-year-old boy from Vietnam born into poverty who was deceived by his “friends” and “kidnapped, raped and trafficked to the UK, and then locked up and forced to grow cannabis” and “when the police found him, he was treated like a criminal rather than a victim.” The men who trafficked him once showed him a “paper that said he owed them £20,000 for his passage to Europe, [and] he was so terrified that he signed it.” They also told him “they knew where his parents lived.” Like the “hundreds of children trafficked from Vietnam every year and forced to work in hidden cannabis farms across the UK” Minh was deemed “valuable” by criminal organizations in cannabis market as he is considered “cheap, expendable, and easy to control and intimidate” in “makeshift cannabis farms in suburban houses, empty flats, deserted warehouses and derelict industrial estates.” Minh spent his days afraid, alone, and locked in a dark room and was given frozen meat to heat up with a microwave. One day he tried to escape but was caught, brought back, and told that “he’d be killed if he tried to escape again.” As this account shows, Minh’s experience was traumatizing and did not end at the cannabis farm as he was convicted of cannabis production and sentenced to eight months in a young offenders’ institution and then moved to an immigration removal center where he was sexually assaulted: “I was very, very scared of these men [the police]. . . . But then I let myself believe that maybe they had come to rescue me. . . . They didn’t ask, so I didn’t say anything, I didn’t know I was allowed. . . . It was like another kind of world. . . I didn’t really even feel human. I understood very quickly that the plants were more valuable than my life. . . . It just felt like my life was over [after the sexual assault]. I just understood that I was not safe anywhere. . . I was very scared of the other inmates and that something like this would happen again. I knew I couldn’t trust the staff there to protect me. . . . After the incident . . . it was like I had split into two different people. . . . I don’t know who I am any more, but maybe there is a way to build a life for myself again . . . Since I left detention I have always felt scared, especially when I thought about how I was trying to fight the police and the Home Office. . . . But I have to try my best to get justice, to take back my life. I have to trust that things can be better.”
**Asset leveraging**
Vignette from the construction industry (UCATT, 2009) Dozens of Lithuanian migrant workers were working on a government hospital construction site in the United Kingdom worth £600 million. The site is managed by a major construction firm, Skanska, who subcontracted to major subcontractors, including a firm called Baris, who subsequently subcontracted to a small dry lining company called Produm, which was the employer of the Lithuanian workers. According to the Union of Construction Allied Trades and Technicians (UCATT), these Lithuanian workers were victims of “a case of appalling systematic abuse of vulnerable migrant workers” by Produm at the hospital construction site. While little to no publicly available first-hand accounts from the workers exist, the following account from UCATT, who unearthed the case and reported it to authorities, reveals that the workers were exploited by their employer Produm, who was also charging them for various assets such as accommodation and tools. Our officer obtained the workers’ pay slips, which revealed that some workers took home just £8.80, after working a 40-hour week. Dry lining subcontracting company Produm employed the dozen Lithuanian workers. The workers were paid below agreed minimum rates for the site operated by main contractor Skanska, they did not receive overtime (some workers worked in excess of 70 hours and took home less than £100) and were charged excessive deductions for rent, tools and utility bills. It is understood that many of these charges were unlawful. UCATT were only able to uncover the extent of the abuse after some of the workers stopped being paid altogether with the company currently owing some workers five weeks’ pay. The workers were initially scared of approaching the union because the company also provided their accommodation.
**Evading legal minimums**
Vignette from the food industry (Lawrence, 2016) Six Lithuanian men who were promised good pay were trafficked to the United Kingdom and exploited by a gangmaster firm to catch chickens on farms, many of which produced eggs for major supermarkets and fast-food chains. As per a judge’s ruling “the men were owed compensation for the firm’s failure to pay the agricultural minimum wage, for the charging of prohibited work-finding fees, for unlawfully withholding wages, and for depriving the workers of facilities to wash, rest, eat and drink.” As 19-year-old Laurynas’s account indicates, being exploited by the gangmaster firm clearly evading legal minimums was horrific: “It’s easy to control people who are scared, controlling your hours, controlling your sleep, controlling your money, controlling everything that you have. . .. We weren’t told how many hours, how many days you need to work, nothing. We were just told you’re going to catch chickens. . .. From the outside, it [the accommodation] didn’t look bad, but when I got inside, I was shocked straight away. But I didn’t have the choice to go back, so I needed to stay. All the people were sleeping in a big bedroom on a mattress on the floor. . .. We were working eight hours on the farm, sit in the van, driving two hours to the next farm, working again about eight hours, sit in the van, getting back, working again, eight or six hours. . .. One of the supervisors beat two people, and once I was nearly beaten after I tried to help one of them get up off the floor. . . He gave me a two-day rest with broken ribs. . .. It’s easy to control people who are scared and don’t know where to go to look for help. . . I never thought that when I left Lithuania that I’d end up like that. I lost trust. I lost trust in people.”
**Workers as consumers**
Vignette from the food industry (Scott et al., 2012) Three Latvian workers were recruited by a gangmaster to work in food production, but ended being underworked and trapped from indebtedness from payments for accommodation (i.e., caravan), transport, unexplained deductions, and borrowed money for basic services such as topping up their phones to call home. One of them was a mother and wife seeking to earn income to be sent back to her family in Latvia, but was unable to do so as they were given so little work, sometimes just one day per week, and was “ashamed” for ending up in such as position, as described in the following account: Almost two weeks without a day off, 11.5 hours per day, after all deductions we received £119. Then when it was quieter I have not received anything. I only got a payslip with all the deductions. I was not even earning enough to pay for accommodation! I was in debt!. . .. Caravans were very crowded, a lot of people, ten people lived in each caravan. . .. In the sitting room, one woman was sleeping on the floor, two of us on the bench, and fourth woman who did not have anywhere to sleep had to wait until everyone had something to eat and then she could move a kitchen table to make some sort of sleeping area. . .. It was beneficial for them to have as many people in the house as possible. The more people were living in the house, the more people were paying for accommodation. Workwise they would give you a little bit of work, so they could get money off us for accommodation. The rest was not important to them. Some people were going into fields to steal potatoes and cabbages because they did not have money to buy food. They did not care about that. The most important for them was to get as many people as possible.

### Risk Reduction Model

In a *risk reduction* model, the deployment of modern slavery serves to reduce the risk that a perpetrator’s illegal enterprise and/or illegal practices will be detected. Specifically, producers in formal and informal industries employ workers in their operations and use coercion and restrictions in workers’ freedoms to reduce labor costs and, importantly, to protect the viability of the firm and its illegal practices or business activities. Yet, our analysis revealed that the risk reduction model is most prominent in two sometimes overlapping contexts—enterprises using *illegal labor practices* within an otherwise legal industry and enterprises engaged in *illegal industries*.

Regarding enterprises using illegal labor practices, we found evidence across both our legal industries—food and construction—that modern slavery may in part serve as means for reducing the risk that illegal labor practices will be detected. However, the deployment of modern slavery as a risk reduction method may not necessarily arise from an employer’s explicit intention to engage in modern slavery but may at times be the result of a downward spiral in labor standards. That is, initial efforts to reduce the impact of labor costs—typically brought on by the cost pressures from buyers further down the value chain—could lead employers to engage in borderline illegal or illegal labor practices to capture more value from workers, such as arbitrary deductions of pay, excessive hours, health and safety violations, or paying below the minimum wage. When employers are subject to some form of external oversight, due either to their geographic location or their position in the supply chain (e.g. they are subject to a retailer’s social audit), this creates a need to conceal said practices to safeguard the firm. This can prompt an employer to engage in further practices that present limits to workers’ freedom, thereby exacerbating exploitation until, in some cases, extreme exploitation crosses the line into modern slavery. At this point, the priority of employers is not only to maintain their minimal labor costs but also to reduce the risk that their illegal labor practices will be detected, creating a vicious cycle of exploitation and concealment. For example, in one report, a homeless man describes how his employer restricted his freedom to conceal exploitative practices:

The work was horrible. The boss was always rushing us and forcing us to do jobs really badly for private customers. . .. To begin with he paid me £20 per day, cash in hand, plus lunch which was a cheap sandwich. . .. The boss was very intimidating and did not like us going off the site on our own. He was always asking where I was going and what I was doing. I had no private life. If I wanted to go to the local shop, he would insist on driving me there and back. He was threatening and would say “I’ll kill you” or “I’ll beat you up,” half joking but in a frightening way. . .. I felt like a virtual prisoner and wanted to escape from the situation but felt I couldn’t. I had become very lonely and depressed and had lost a lot of confidence. I was ashamed of what was happening to me. (Elliot & Lucio, 2011, p. 17)

Our analysis revealed that, in addition to coercion to force workers to conceal employers’ practices, employers would also deliberately target highly vulnerable workers to reduce risks. They typically target workers who are illegal immigrants, lack skills and a basic understanding of the local language and norms, and come from countries where there is a distrust for authorities. These factors all contribute to a critical layer of opacity that impedes the detection and prosecution of exploitative value-capturing practices in businesses. For example, one of our social auditor informants described a case of a food warehouse in which a group of Romanian women were intentionally recruited into forced labor because they did not speak English well and would not have the power to ask questions or seek help. Thus, it is often difficult for workers to create social support networks, even if they are not confined to a site. Moreover, as one informant explained, negative perceptions of authorities function as a safeguard to reduce risks of detection:

The huge problem in trying to expose wrongdoing in this whole area of exploited workers is that they’re all afraid so they won’t come forward. Particularly as many of them are Eastern Europeans. . .where the authorities are the enemy and if you complain you’ll be beaten up. It really is like that and so they are terrified of authority and they don’t want to, you know they don’t trust the system. They don’t want to speak out because they’re afraid so that makes it much easier for the employers to exploit them. (Journalist and Consultant, Interview).

Second, we found that the risk reduction business model of modern slavery could be used by firms operating in informal or illegal industries to reduce the risk that their illegal enterprises will be detected. Specifically, illicit enterprises are vulnerable to the risk that their workers will engage in whistleblowing and report their illegal activities. Our analysis indicated that modern slavery practices enable cannabis producers to reduce their risk of being detected, thus protecting the viability of their enterprises—of course, at the expense of considerable hardship for workers.

The perpetrators in this model were typically reported to be organized criminal gangs, usually of Vietnamese or Chinese origin. We found that the victims would usually be workers of the same nationality or ethnicity as their perpetrators who were trafficked in from overseas. Risk reduction is, on the one hand, achieved via coercive means such as physical constraints and threats by an employer to force workers to hide their practices. For instance, in the case of R v N; R v LE (2012), the appellant, who did not speak English, was found to have been locked up in a cannabis farm with brick-covered windows and doors, guarded by gun-carrying security guards. He was found to be unpaid and was threatened with death upon attempting to escape. On the other hand, by bringing in illegal workers to work as “gardeners,” employers in cannabis production can also reduce the risk that their illegal activities will be discovered. In particular, because illegal workers possess high vulnerability, and may be confined to a site, they will be unlikely to report their employer’s illegal enterprises to the authorities, thus reducing risks. This is particularly the case for children in situations of forced labor because even if they are caught, they are unlikely to be prosecuted, thereby further reducing risk as explained by this informant:

There has been probably an increasing trend to use younger people. . .. I think maybe they perceive that there’s maybe less risk with children, children will then not be prosecuted, not go into the criminal system and may not say anything because they’re not being prosecuted and they may not identify who the traffickers are. But whatever model they use it will be to minimise the risk to those who are ultimately responsible. (Director, Consulting Firm, Interview)

The risk reduction model possesses a key distinction in comparison to traditional slavery business models in that new linkages are formed between restrictions on freedom and value creation and capture. Rather than such restrictions being solely about labor cost reductions, they also improve the effectiveness of, and reduce the costs of, concealment of illegal value creating or capturing practices by businesses.

### Asset Leveraging

In an *asset leveraging* model, a producer’s use of modern slavery entails new activities that leverage existing assets to generate additional revenue streams. However, it is important to note that revenue generation co-exists with cost minimization, and the two components are not easily disentangled from each other. Nonetheless, our analysis indicates that revenue generation is often achieved by charging workers for ancillary services, and is more commonly found in contexts where profitability, or the potential to capture value from the core business activity, is relatively low—again, often as a result of value capture from firms further along the supply chain.

For instance, in farming, the economic logic in many developed countries is that small operations struggle to survive in the face of consolidation, which creates larger and more powerful competitors that can reap economies of scale. Food processors and retailers are also mainly large players that can dictate prices through the supply chain, which leaves farmers at the bottom of the supply chain with narrow margins. Typically, small farmers running legitimate businesses will look to capitalize on their assets by expanding their portfolio of operations and engaging in creative diversification into value-adding processes such as packaging, tourism (e.g., operating tours or a bed and breakfast), retail (e.g., selling at a farm or farmers’ markets), or energy generation (Morris et al., 2017). However, small farmers using forced labor will leverage assets in two ways: (a) leveraging the organization’s assets and (b) leveraging workers’ assets.

#### Leveraging the organization’s assets

When producers leverage their own assets, (e.g., a barn or caravan), they drive additional revenue by charging their workers for ancillary services, typically accommodation, but sometimes meals and transportation. Importantly, these charges will often be involuntary or hidden for workers, meaning that they need bear little resemblance to market rates and quality standards. Thus, firms—usually those operating in legal industries, but with little opportunity for value capture because of their supply chain context—resort to overcharging workers for sub-par goods and services for which the workers have no other options. As one informant, a public-sector director explained, “what people tend to do is provide the transportation and then charge for the transportation. The transportation might not be safe and it might be uneconomical. You know, an excessive charge” (Director, Governmental Organization, Interview).

However, the way that producers collect revenue from ancillary services makes it difficult to disentangle revenue generation and cost minimization. For instance, some employers will pay workers a legal, albeit minimum wage, but make them immediately pay for ancillary services, which allows firms to operate within legal boundaries, as this informant explained:

[I]f you provide accommodation and you charge for it and deduct the amount of the charge from the wages, you can breach the national minimum wage. If, conversely, you pay the worker everything, give them the money, then from that, either on the day they get on the bus or at any other point, say to them, “right, well you owe us £25 for that,” and the worker hands over £25, although it’s the employer that’s running the accommodation and providing the work and paying and taking £25, because the payment is taken from the worker’s hand, not from the worker’s wage packet, it doesn’t breach national minimum wage. (Director, Governmental Organization, Interview)

Indeed, some workers might never see any or most of the money that they earn due to automatic (and often fraudulent) deductions. For example, a union representative recalled that they found payslip evidence that migrant workers on a hospital project were taking home £8.80 ($10.67 USD) a week after deductions for accommodation and tools (Representative, Trade Union, Interview). With this method, firms essentially charge workers to generate revenue that negates or covers a large portion of wage costs, creating revenue streams that recoup or cancel out wages. Viewed another way, firms are repackaging a labor cost reduction to give workers the impression that they are making a wage.

Another way that producers generate revenue with ancillary services is via indebtedness or debt bondage. The leveraging assets model is most likely to be used where workers are already extremely vulnerable; those with little capital or existing debts (such as those incurred through recruitment or migration) are especially targeted. Workers can be forced into debt to the producer in this model when workers do not make enough income to pay for ancillary services. This may be because a producer charges excessive prices and/or does not offer sufficient wages to pay off costs. We found that this form of indebtedness may be leveraged to enable an employer to extract more work out of a worker and decrease per unit costs, a reminiscence of traditional slavery. Thus, workers repay debt through labor and a producer leverages workers, as human capital, to generate more units of production.

Charging for ancillary services creates greater potential for opacity in the debts accrued by workers, which provides increased control for employers (Crane, 2013). To maximize returns on ancillary services, producers impose obstacles to workers leaving, such as threats, withholding wages, or confiscating documents, as this testimony from a Latvian farmer worker in one of our archival sources illustrates:

Oh, they took my passport and after three weeks they did not return my passport. I went to ask for it. . .. They always were coming up with good excuses. . ., I was trying to get my passport back for a year, but they would not return it to me. It was until they found out that someone was coming to inspect the farm. That same evening they returned passports to all of us. . . .We had wanted to leave for some time, but we could not without our passports. We realized that it could not be like this, that we work hard and do not earn much. We could not go anywhere without our passports, but when they returned our passports three of us (me and two friends who I met on the farm) we run away from the farm. We owed the farmer about £100 for the caravan and food. We did not want to work there, so we run away from the farm. (Scott et al., 2012, p. 54)

#### Leveraging workers’ assets

Producers can also generate additional revenue via new activities that leverage a worker’s assets, rights, or privileges. For instance, modern slavery enterprises, especially those that are operating illegally or informally, sometimes generate revenue via identity and benefit theft. Such employers confiscate workers’ identification documents, which helps create dependence and control while restricting freedom of movement. Employers then use these documents to make fraudulent benefit and other claims, force workers to make claims whether they are entitled to them or not, or sell the documents to generate revenue. Workers typically see little or none of the proceeds since any revenue generated is directly captured by employers. As one report put it:

Victims are brought to the UK with the promise of jobs, have benefits registered in their names and are then left destitute, without jobs or homes. It is estimated by police that millions of pounds are being removed from the UK in the form of benefits paid to individuals who do not receive any of the money. (Slavery Working Group, 2013, p. 47)

Victims in this model are usually vulnerable workers that typically have some status within the relevant welfare regime that can be exploited. This includes those who are homeless or suffering from mental health or addiction problems, or those that (in the case of the United Kingdom) are European Union nationals unaware of or unable to exercise their rights.

The asset leveraging model has parallels with traditional slavery business models of the Antebellum South as it focuses on maximizing value from a fixed set of resources. However, whereas most Southern planters focused on extracting as much labor from their workers to attain economies of scale in their usual business (Wilson, 1996), modern slavery enterprises engage their workers in a wider range of activities that enable the organization to capture value from their exploited workers.

### Evading Legal Minimums

We now turn to the novel business models of modern slavery deployed by labor market intermediaries, which have both similarities and differences in comparison to producers. Examples of intermediaries that might be engaged in modern slavery practices are agencies, recruitment agents, labor providers, and gangmasters. More specifically, intermediaries (whether involved in modern slavery or not) are understood as organizations that supposedly create value by conducting activities such as finding, selecting, hiring, deploying, training, firing, or administering workers for producers more efficiently or effectively than they could achieve themselves (Bonet et al., 2013).

In the *evading legal minimums* model, the key driver is the intermediary’s need to reduce labor costs just as it is for many producers and indeed as it was in traditional slavery. However, the involvement of intermediaries as new actors actually deploying slavery, rather than just trading the enslaved, precipitates new activities and new forms of value capture.

Intermediaries have little control over the price charged for labor as it is typically dictated by clients. A senior executive at a recruiting agency suggested that, as a result, clients help drive unscrupulous agents towards illegality to lower costs:

So I put in my unit price for finding these people. . .familiarising them, preparing them, getting their visa, transporting them, making sure they’ve got decent accommodation, and boarding them. And then making sure that, over the two-year assignment, they get treated correctly, then taking them back to their home country. So, my cost would be $500 to provide you, Mr. Customer, with that person. A local agent, [the cost would be] between $50 and $100. Guess which bid the customer accepts? The agent that is operating illegally. They will not take my cost. It is five times higher than somebody else’s because I’m acting ethically. (President, Corporation, Interview)

In seeking to reduce labor costs to increase their margins, agencies will thus sometimes resort to illegal ways to capture value. As an NGO respondent (Interview) noted, “the further down the supply chain you go, the lower the profit margins are, and therefore the way in which you can maximize your profits is by cutting corners.” This pressure on margins triggers intermediaries to respond in creative ways to reduce labor costs.

First, some agencies will minimize labor costs by outsourcing or *subcontracting to other intermediaries*. Subcontracted intermediaries will typically operate with lower labor costs, and these may subsequently outsource to other providers, including informal and independent operators at even lower cost. These more informal or unregulated outsourced intermediaries serve as key partners to producers and intermediaries because they offer the essential resource of labor through unique channels, and usually at very low cost. While outsourcing does not mean that exploitation will occur, the use of informal and independent operators creates the conditions where such forms of exploitation are more likely thrive (Crane et al., 2019). As an NGO informant explained:

In recent history there has often been concerns raised about triangular relationships between the worker and the agency and employer, but I think that is getting increasingly more complicated and I am aware that there can be four, maybe five different actors involved in that relationship. That added complication certainly facilitates exploitation because it is much harder to really understand the terms and conditions that somebody’s employed and contracted under. (Manager, NGO, Interview)

Informality in labor provision often entails the use of vulnerable foreign, migrant, or smuggled workers who have already been exploited, deceived, or coerced in forced labor arrangements in their home countries, as this informant explained:

Often these are perfectly ordinary young people who have seen an opportunity to get work in the UK who are recruited by unscrupulous people in their own country and they get told, “Here’s $500,” or whatever it is, “Go to this address in the UK and they will give you work.” And then the people arrive, having been already hooked into the system and end up in a forced labor situation. (Director, Multi-stakeholder Initiative, Interview)

A second way that intermediaries will evade legal minimums is through new activities that *exploit regulatory loopholes* to reduce costs. This is more likely to be associated with formal intermediaries targeting vulnerable workers, often across borders. For example, the use of fraudulent self-employment schemes enables workers to be officially designated as self-employed, while still effectively controlled by the intermediary. This enables the intermediary to bypass regulations regarding pay, working conditions, benefits, and tax obligations to reduce the costs of employment. In other cases, intermediaries may employ workers in one country with minimal labor standards but deploy them in the United Kingdom to avoid UK protections. These intermediaries may “push the law to the absolute limit and often break it, but in a way they think they can get away with” (Journalist and Consultant, Interview). These activities will not necessarily lead to extreme exploitation, but they do provide the conditions under which a business model for modern slavery can be deployed.

In turn, while intermediaries indeed played a key role in traditional slavery, the evading legal minimums model represents a key distinction in how intermediaries have evolved. Rather than being restricted to the function of supplying the enslaved to enterprises (as was common in traditional slavery), some intermediaries have now assumed the role of slavery enterprises—that is, they are the actors actually deploying modern slavery. As such they are engaging in a new set of activities that ostensibly create value for producers and other intermediaries by offering low-cost labor provision that avoids statutory labor standards but at the cost of extreme worker exploitation.

### Workers as Consumers

Lastly, in a *workers as consumers* model, rather than just earning a margin on the revenue from supplying labor to clients, the intermediary also generates revenue by providing ancillary services such as basic living services including accommodation and food, as well as services that are necessary for work such as transportation to and from company housing to work sites, or the use of company tools. While this has much in common with the asset leveraging model used by producers in that it involves the generation of revenue from exploited workers, the business model for intermediaries has some key features.

These differences are most evident where intermediaries create new linkages between revenue, debt, and labor. Intermediaries will use debt to gain control over workers so that they can exploit them (Friebel & Guriev, 2006). The sale of ancillary services is used both to generate and sustain indebtedness, and thus control and obedience, as well as to generate revenue, as this informant explained.

I mean, the whole kind of control method is debt, isn’t it? You know, from the very start. . .. The whole thing is that the person is in debt from the outset because they pay for their travel, they pay for any visas, whether they need them or not, they then go into the work situation. And there’s two models. One is you can never pay back the debt, therefore you need to work. So, one model is you always owe so therefore you work and therefore you take the exploitative situation because you need to pay back and you may never pay back because it may be interest, you may move on, you may have add-ons. The other model is you don’t get paid. (Director, Consulting Firm, Interview)

In some cases, this may even give rise to a seemingly paradoxical situation of intermediaries deliberately *underworking* their forced laborers. In this model, the intermediary intentionally takes on more workers than it needs for the work it expects to get from its clients. This oversupply leads to underemployment for the workers involved, who receive perhaps only a few hours or one or two days of work a week. Meanwhile, the intermediary will continually charge these workers for accommodation and other ancillary services at a rate that exceeds their earnings, thus forcing them deeper into debt to the intermediary, as described here:

So it’s quite literally people being told well, you come to the UK, we’ll lend you the money or if you get here we’ll provide you with accommodation and wages, work and then when they get here they deliberately don’t give them any work to do. Say, look in another two weeks’ time, three weeks’ time we can give you work. At the moment, there’s none but don’t worry about it, you can stay in the accommodation we provided. There’s a bit of money so you don’t starve, pay me back when you start getting your wages. So, they sound very nice and reasonable but the thing is to build up this bondage so they can’t just walk away. (Policy Officer, Trade Union, Interview)

To service this debt, workers may secure outside income from family members abroad or instant loan services, which enables intermediaries to generate additional revenue. In other cases, workers will continue to accumulate large amounts of debt, usually with undisclosed premium interest rates, that they cannot repay. Consequently, workers are prevented from gaining financial independence and become increasingly susceptible to continued exploitation. We label this model “workers as consumers” because consumption of ancillary services is central to the operation of this approach, while in the “asset leveraging” model it is typically just an add-on. As a trade union informant suggests, charges for ancillary services can be sustained even when workers are not being productively deployed, thereby ensuring a constant stream of revenue:

But you get examples where they’re being charged even when they’re not being taken to work and the employer will say, yes but I’ve had to buy this van. I’m still paying for this van. Just because I can’t give your work at this moment, I don’t stop paying for this van so you’re going to help me pay for this van. (Policy Officer, Trade Union, Interview)

Thus, unlike intermediaries that focus on minimizing the unit cost of labor, these intermediaries will be less concerned with maximizing its margin charged to clients, and more concerned with maximizing the number of workers under its control and maximizing the margin earned from services supplied to its workers. Therefore, the workers as consumers model of modern slavery is probably the most distinct from traditional slavery. Rather than over-utilizing workers, intermediaries seek to grow their stock of human capital, but underutilize workers. Nevertheless, like the other models, this model entails considerably hardship and unfreedom for workers, who are indebted, subjected to exploitation, and face considerable constraints to their freedom of movement.

## Discussion

In our findings, we identified and dissected four business models of modern slavery that enable enterprises to profitably exploit vulnerable workers but also possess novel distinctions from the business models of traditional slavery. These novel models suggest that both continuities and divergences from the business models of traditional slavery persist in contemporary society. In this section, we explain these continuities and divergences, and explore their implications for our understanding of the business of modern slavery and our understanding of business model innovation more broadly. We conclude by outlining the implications for policy and practice.

### Continuities with Traditional Slavery

In terms of continuities with the business models of traditional slavery, several key features continue to be manifest in modern slavery, and indeed build the foundation of the modern slavery business models that we identify. Two are particularly worthy of note: an economic rationale and a reliance on coercion.

#### Economic rationale

Just like traditional slavery (e.g., Fogel & Engerman, 1980; Metzer, 1975), modern slavery continues to be motivated by an economic rationale, often but not exclusively as a way to achieve cost minimization from the “cheapness of the labor” (Williams, 1944, p. 19) or compressing labor costs below the legal minimum. As was the case with Southern plantations, in modern production firms in labor intensive industries, such as construction and agriculture, as well among labor intermediaries in most industries, wages and other labor costs constitute the major driver of profitability. These variable costs make up such a large proportion of total costs that they are the most viable levers for increasing profitability, especially given that most value tends to be captured elsewhere in the supply chain. Therefore, enterprises may look to minimize labor costs through modern slavery rather than other forms of innovation to undercut competition and maximize profitability (e.g., Miller & Smith, 1997), and in doing so, they evade laws and impose abusive conditions onto their workforces. However, as we show, focusing only on labor costs misses an essential part of the picture. It is important not to ignore risk reduction and revenue generation that provide an economic underpinning for new business models in addition to labor cost minimization as we discuss further in relation to divergences.

#### Reliance on coercion

Coercion as an enforcement mechanism also continues to persist as an essential element sustaining slavery business models. This can be achieved through the “threat of physical punishment” (Ransom & Sutch, 1977, p. 20) or physical constraints by an employer on a laborer. While the physical shackles of the past have disappeared in many contexts, new business models continue to rely on threats, fear, and deception to function.

### Divergences in the Business Models of Modern Slavery

Despite these continuities, there are also important divergences, which can be effectively interpreted as “innovations” (albeit oppressive ones) in the business models of modern slavery in the form of: new activities, linking activities in new ways, and changing which actors perform activities (Amit & Zott, 2012)

#### New activities in modern slavery

One critical change in activities that enables new business models of modern slavery is a shift from upfront purchase of enslaved people to new activities involved in exercising control over workers. Modern slavery largely involves the management of debt-labor contracts, enforced by coercion and threats, rather than capital purchases enforced by legal institutions (Crane, 2013). As LeBaron (2014, p. 766) puts it, “debt functions as a lever into the labor market for those with few alternatives, a form of coercion that precludes exit from it, and a form of control that allows employers to maximize their exploitation within it.” Further, as Bales (2004, p. 25) has argued, this marks a shift “away from ownership and fixed asset management, concentrating instead on control and use of resources.” Thus, whereas a feature of slavery in the Southern plantation was the transformation of labor costs from a variable cost to an upfront fixed cost (Anderson & Gallman, 1977; Metzer, 1975), a feature of modern slavery is the shift back to variable costs where laborers might receive some kind of wages or remuneration (albeit limited, recouped, or conditional) and are under control for shorter periods.

Therefore, the business models of modern slavery often include new activities that help to generate value for employers through worker exploitation in a shorter period. Such activities go beyond the traditional model of slaves participating in production and carrying out domestic services. Instead, some employers generate revenue from the assets of their workers, while others use their workers to minimize the risk of detection of illicit practices. In turn, the business models of modern slavery are not just based on minimizing labor costs and solving a labor supply problem as is typically assumed for traditional slavery, and as much of the modern slavery literature would suggest (e.g. Marschke & Vandergeest, 2016; Phillips & Mieres, 2014).

#### Linking activities in new ways in modern slavery

The linking of activities in new ways, or what Amit and Zott (2012, p. 44) call innovations in the “structure of an activity system,” is also present in some of the new business models of modern slavery. For example, our research uncovered creative ways of integrating employment relationships with consumption relationships. In some cases, modern slavery operators take advantage of their control over workers to turn them into captive “customers” for a range of ancillary services, and often charge workers usurious rates for these services. These services might vary, but the most common are accommodation, food, transport, and immigration services. While the provision of such services might also be a feature of free labor relationships, as well as traditional slavery—Southern slave owners provided “the food, shelter and clothing necessary to keep the slaves healthy and hardworking” (Ransom & Sutch, 1977, p. 7)—in modern slavery, workers typically have little option but to pay for these services and will often not know whether or how much they have been charged. The innovation here is that employers use a forced labor employment relationship to acquire a monopolistic position in supplying ancillary services and can charge prices higher than market rates. These can be an important and relatively stable revenue stream for perpetrators. Charging for these ancillary services does not just generate revenue for perpetrators, but also enables them to create greater indebtedness (i.e., debt-bondage) and greater vulnerability. Thus, employment and consumption are linked in new ways to create a business model of modern slavery more attuned to revenue generation than simply labor costs.

There are, of course, parallels here with how consumption, work, and debt are interrelated at the system level. That is, as inequality has surged within the contemporary global capitalist economy, many households have increased their labor market activity (such as by additional family members entering the labor force) to maintain consumption levels. However, as access to wage labor and decent work is highly limited in some contexts, gaining access to jobs domestically and abroad often requires taking on debt to intermediaries (LeBaron, 2020). For instance, one recent study estimates that migrant workers around the world pay US$10–20 billion a year in recruitment fees, and considerable portions of those fees are paid for through debt (Martin, 2017). Debt is becoming an increasingly common strategy to maintain class status and consumption levels across the skilled and “unskilled” ends of the labor market, and across the global North and South (Graeber, 2011; Soederberg, 2014). However, while debt functions as a mechanism of labor market control and discipline for large swathes of the labor market, it renders workers at the bottom end of the labor market particularly vulnerable to forced labor, due to the lack of alternative means to obtain their subsistence (LeBaron, 2014).

#### New actors in modern slavery

Plantation owners were the key actors deploying the enslaved in traditional slavery—they forced people to work and made enslaved labor a key resource in the operation of their businesses. In some modern slavery businesses, plantation owners have been replaced by other broadly similar producers, such as farmers, factory owners, or mine operators (Bales, 2004). A key difference, though, is that producers and those they exploit are not necessarily divided by race, or even at times nationality, as they were in the Antebellum South. However, racial discrimination does remain a key determinant of exploitation—albeit very much along with other markers of discrimination, such as gender, ethnicity, caste, and various other social categorizations that are used to create and justify exploitation (LeBaron et al., 2018). Therefore, although the form of discrimination may have changed, it is “rooted in the very same logics” as the slavery of the Antebellum South (LeBaron et al., 2018).

In addition, other actors need also to be considered, which has tended to be overlooked in existing accounts of modern slavery, where “employers” are often represented in quite homogenous terms. The main development here is that in contrast to the Antebellum South, labor market intermediaries also play a key role deploying modern slavery and so it is the business models of intermediaries that need also to be addressed.^4^ These individuals or organizations, who are players in business value chains or activity systems (Amit & Zott, 2012), “mediate between individual workers and the organizations that need work done, shaping how workers are matched to organizations, how tasks are performed, and how conflicts are resolved” (Bonet et al., 2013, p. 341). So, intermediaries are not directly engaged in production, but provide the key resources of labor and labor-related services to producers, making them essential partners in producers’ own business models (Osterwalder & Pigneur 2010).

While much traditional slavery relied on a process of recruitment, movement, and deployment where slaveholders were typically distinct from slave traders (Baptist, 2014), many intermediaries are now not only traders but also play a much more active role in deploying and extracting value from enslaved people, often unbeknownst to producers themselves. Therefore, while we tend to think of intermediaries as being middlemen between producers and those enslaved, involved principally in trade, they now often retain a relationship with forced laborers, and may indeed be the actor exercising control over them. This is part of a broader shift from a dyadic employment relationship between employer and worker to a triangular relationship where employer roles and responsibilities may be blurred across different actors (Bonet et al., 2013; Davidov, 2004; Vosko, 1997). In the context of modern slavery, we demonstrate that distinct business models have arisen for producers and intermediaries in deploying forced labor. Although intermediaries were clearly present in traditional slavery, their involvement in the business models of modern slavery clearly represents a new actor role with associated new activities and activity linkages.

### Implications for Research

Our analysis has important implications for research on the business of modern slavery and on business models more broadly.

#### Business of modern slavery

Our study has implications for the business of modern slavery literature. First, as stated earlier, this literature tends to only touch lightly upon the mechanics of how businesses actually operate. In the portion of this literature anchored in development studies and political economy (Barrientos et al., 2013; Phillips, 2013), there is often an overwhelming focus on the role of big brand and multi-national companies like Nike or Walmart in shaping the conditions for labor standards within global supply chains. While the role of such companies is no doubt important, especially in creating the conditions for the emergence of the business models we identify here, the focus on the top of the supply chain has meant that the sub-tiers of production, where modern slavery actually tends to thrive, has been understudied. Our research highlights the importance of understanding the businesses at the bottom of such supply chains, and especially in pinpointing the value of modern slavery to their operations and how they profit from it.

Clearly the business models of multinationals and the business models of modern slavery enterprises that we focus on here are not unrelated. When a multinational shifts its business model to seek the highest flexibility and lowest possible cost through outsourcing, for example, this can set in train a drive to increase flexibility and reduce cost throughout the supply chain. As a result, modern slavery enterprises may emerge to help facilitate this, and they in turn may need to adapt their business models—to generate revenue or reduce costs—in order to succeed in doing so. Therefore, the business models we have identified can also at times be part of broader business models of multinationals, and indeed can be a key aspect in making them feasible.

**Figure 2.**
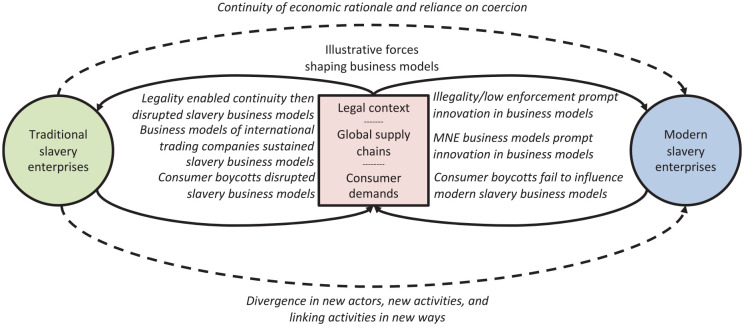
Traditional and modern slavery business models.

We illustrate this in Figure 2 where we summarize the continuity and divergence in traditional and modern slavery business models, and illustrate some of the economic and legal forces that shape these business models.

What this means is that the business models we identify—as with the business models of traditional slavery—do not appear out of a vacuum, or simply as a result of individual criminal intent, but are embedded within a broader capitalist global economy that makes their emergence somewhat predictable (see LeBaron et al 2018 for an overview and typology of how capitalism gives rise to both a supply of vulnerable workers and business demand for forced labor). As Banerjee (2021, p.415) argues,

[S]lavery is an abhorrent practice and while it is technically a crime under international law, it is important to realize that it remains a viable and profitable management practice for business. Modern slavery, far from being an aberration, is a logical outcome of the way our political economic system is organized and its historical origins in the colonial enterprise.

Indeed, evidence suggests that most identified cases of modern slavery demonstrate connections—either through economic or social ties—to a range of legitimate businesses (De Vries, 2019).

In Figure 2, we show that global economy forces such as the legal context, global supply chains, and consumer demand help to explain why both the business models of traditional slavery enterprises were sustained and then disrupted, and why innovation in business models has been prompted in modern slavery enterprises. These are clearly not the only such forces that are relevant, but we include them for illustrative purposes of the more general point. Specifically, the legal context helped sustain earlier business models by establishing the legality of slavery but then the forces of abolition severely disrupted these models from the 19th century onwards. Likewise, for modern slavery, illegality coupled with poor enforcement has been a major influence on the type of business models that have emerged. Similarly, just as the global slave trade and the demand for commodities from international trading companies sustained traditional slavery business models, so too do the business models of present-day multinationals prompt innovations in modern slavery business models. Consumer demand, meanwhile, played an important role in disrupting traditional slavery business models in the form of boycotts, while current consumers seem unwilling or unable to effect similar change in modern slavery business models (Smith & Johns, 2019). We will return to these forces in our discussion of implications for policy and practice below.

It is also evident, however, that not all modern slavery enterprises are embedded in the supply chains of multinationals, or subject to the demands of consumers in the global North. The business models we identified that are least likely to be such—the illegal markets variant of the risk reduction model and the workers’ assets variant of the asset leveraging model—demonstrate that evolutions in the business models of modern slavery can also occur among informal and illegal enterprises whose products and workers do not end up in large companies. This highlights the importance of identifying how modern slavery enterprises make money and why they resort to such practices as part of a business model because the profit imperative is present irrespective of the degree of formality of legality of the business—even though the practices and contexts may vary. In illuminating these variations of modern slavery enterprises, we broaden the discussion in the literature to discuss a broader range of business settings linked to modern slavery.

In the popular literature on modern slavery (Bales, 2004; Bowe, 2007; Kara 2009), the business of modern slavery tends to be presented in a homogenous and overly simplistic way. Our research contradicts some of the literature’s characterizations of the business of modern slavery, such as that victims are unpaid. For example, Kara (2009, p. 11) defines modern slavery in the sex industry as “the violent coercion of unpaid sex services”. It also shows that there is significant variation in the role that modern slavery plays within a business model, and how a business tends to create and capture value through slavery. These variations have not been recognized in the modern slavery literature, where businesses have tended to be studied in highly superficial ways and where scholars tend to assume that modern slavery occurs randomly within the economy, and it is thus not possible to systematically map and understand associated business models.

#### Business models

Our study offers new insights into the dark side of business models and their evolution over time. Although research has increasingly focused on how new business models can benefit the environment and society (Massa et al., 2017), there is scant research focused on understanding how changes in business models can explicitly harm individuals or society (Martí, 2018). However, with growing attention to the potential negative effects of business models in the digital, sharing, and gig economies (e.g. Friedman, 2014; Todolí-Signes, 2017), our study seeks to make new connections between the business model literature and research on the broader pathologies and social harms of contemporary organizational practice (Linstead et al., 2014).

Critics of businesses involved in slavery as well as other less extreme forms of worker exploitation, including those in the gig economy, tend to blame “the business model”. For instance, Aloisi (2016) examines the “the underlying business model” (p. 64) of the sharing economy through case studies such as Uber and Amazon Mechanical Turk to show how it exploits and reproduces precarious employment. Likewise, Ewart-James and Wilkins (2015) associate the exploitation of migrants in the hospitality industry with the “low cost/low value business model” adopted by the industry. More broadly, companies as diverse as Apple (Lehman & Haslam, 2013) and Walmart (Sethi, 2014) have seen their core business model identified as the cause of worker exploitation and other social ills.

While such studies have provided valuable insights on exploitative business models at both firm and industry levels, they have tended to ignore smaller firms at the bottom of the supply chain, and how their business models have evolved and adapted to these broader changes. Moreover, their focus on a specific, single business model suggests there is a single underlying rationale for how organizations create and capture value that leads to negative social outcomes. Our study demonstrates that an analysis of oppressive business model changes can unpack these assumptions and contribute new insight into the diversity of such models and how, in distinct ways, the new actors, activities, and linking of activities that constitute them give rise to different manifestations of exploitation and social harm.

### Implications for Policy and Practice

Our analysis also has important implications for policy and practice, particularly as it pertains to detecting and remedying modern slavery in the United Kingdom, and in the global economy more broadly. We believe that confronting the business models of modern slavery can and should play a critical role in helping to reduce the incidence of extreme exploitation, and in developing less exploitative ways of doing business.

#### Policy implications

At present, in most jurisdictions, there are sparse resources for labor enforcement, inspection of workplaces is minimal, and policing of extreme forms of exploitation is hampered by problems of detection of crimes and of identification of victims and perpetrators (Crane et al., 2019). This creates a context wherein business models configured around modern slavery can be enacted with widespread impunity, in spite of the fact that forced labor is a crime. Our findings can be used for law enforcement by profiling different forms of perpetrators based on their business models, thereby enabling “follow-the-money” type crime control strategies (see Naylor, 2003). Specifically, returning to Table 2, there are particular victims and perpetrators that are more likely to be associated with particular modern slavery business models, which can enable enhanced profiling and better focusing of law enforcement resources. For example, law enforcement agencies targeting labor intermediaries involved in modern slavery might choose to concentrate on those operating the workers as consumers models—and therefore their attention should focus on investigating deductions from migrant workers’ wages as an initial starting point to “follow-the-money.”

In addition, understanding the business models of modern slavery can help in devising better forms of business regulation, licensing, and taxation that might create additional economic disincentives for perpetrators. The challenge here is to find ways that would disrupt existing business models. For example, one potential pathway for new regulation could be the requirement for additional licensing requirements for organizations seeking to provide accommodation or transport services. Providing such licensing was adequately enforced, this could help in preventing some enterprises from perpetrating modern slavery by making it more difficult to operate either a workers as consumers model for intermediaries or an asset leveraging model for producers. Another potential pathway could be new legal requirements for landlords to conduct regular property inspections. Such an intervention could significantly shift the risk calculus of criminals thinking of using forced labor in cannabis production. That is, if cannabis producers knew that landlords were compelled to inspect their properties, the economic benefits of using forced labor to prevent detection might be reduced sufficiently.

Relatedly, although current approaches to tackling modern slavery are hampered by assumptions that it is a hidden crime, our research suggests that it is possible to pinpoint common characteristics of organizations that use modern slavery, and thereby target policy and policing interventions, as well as victim support systems, accordingly. Potential indicators of modern slavery suggested by our analysis would include the following: (a) high levels of labor intermediation in the context of low wage jobs on or around the minimum wage; (b) low levels of value capture at specific levels of the value chain (thereby precipitating the cutting of corners or provision of additional revenue generating activities); (c) workers required to purchase high priced ancillary services; and (d) more common indicators such as passport and other identity document retention. With the proliferation of policy and legislation targeting modern slavery and related human rights issues in supply chains, such insight can help policy-makers better develop and revise their efforts to remedy modern slavery.

Ultimately, our research also highlights the importance of social protection for low-wage and migrant workers, and for ensuring robust forms of labor law enforcement that prevent businesses from compressing labor costs below minimum wage. At present, modern slavery legislation is primarily focused on increasing transparency in the supply chains of large companies. However, this approach has a number of in-built structural limitations for dealing with the modern slavery business models identified in our study. First, some of these enterprises do not even operate in the supply chains of large companies and so will be overlooked by the legislation. Second, the legislation is based on an as yet untested assumption that final consumers will actually act on information about slavery in supply chains—even if it were reliably and accurately provided. As Smith and Johns (2019) argue, moral indifference may be a better explanation for a lack of consumer action on the issue of modern slavery than ignorance alone. This is supported by research from Carrington et al (2018, p. 14) who found that rather than acting on their concerns about exploitative practices, “more commonly, consumers came up with a series of justifications or neutralizations that allowed them to remove any sense of personal guilt or responsibility in relation to modern slavery.” Hence, although consumers were effective in disrupting the business models of traditional slavery, in the context of modern slavery, regulators would be better advised to directly protect workers in exploitative situations rather than trusting that market mechanisms will work through the power of consumer activism.

#### Practitioner implications

For business practitioners, our findings also provide new insight on how to protect their own businesses from the taint of slavery or how to address the competitive threats posed by competitors using modern slavery to undercut them. Regarding the former, as suggested earlier, our analysis helps identify new risk factors that are likely to be associated with modern slavery enterprises that might be in companies’ supply chains. These include intermediaries that have additional revenue streams or the use of workers with unsustainable consumption relationships with their employers. These risk factors can potentially be identified by companies through more sophisticated social accountability processes such as worker voice mechanisms (Gunawardana, 2014) that can then prompt appropriate interventions. Again, by identifying in our analysis which workers are likely to be vulnerable to which business models, companies can more effectively identify potential victims and determine what information they might prioritize in collecting from and about them.

We also provide a platform for more accurately identifying why and how modern slavery enterprises in a firm’s supply chain engage in extreme exploitation as a way of sustaining their businesses. This can be used by firms to better identify where in their supply chain they should target social responsibility initiatives aimed at tackling modern slavery. In particular, companies should consider going beyond simply auditing working conditions to developing initiatives that are designed to enhance the value available to suppliers most at risk of crossing the line into extreme exploitation. As we show in Figure 2, the business models of slavery enterprises are shaped at least in part by the business models of corporate actors forcing low prices through the supply chain in order to enhance their own value capture. As a result, there is a case to be made that brands may be complicit in many of these instances of business model innovation by modern slavery enterprises. It is only by reducing the pressure on such suppliers and ensuring that there is more equitable distribution of value through the supply chain that the business models we identify can be effectively confronted by corporations.

## Notes

1 While slavery was made illegal in much of the world during the 19th century, legislation to prohibit specific forms of slavery such as bonded labor were typically instituted much later. For example, legislation addressing bonded labor was introduced in India in 1976, in Pakistan in 1992, and in Nepal in 2000 (see Quirk 2008).

2 Most notable here has been Kara’s (2009, 2017) work. Such work has been critiqued several times in the academic literature (e.g., Agustín, 2012; Andersson et al., 2019).

3 See the ILO Forced Labour Convention, 1930 (No. 29), available at https://bit.ly/2kvyezD

4 Labor market intermediaries have also earlier played more direct roles in manifestations of slavery after abolition, including for example the padrones who exploited immigrant workers in North America in the late 1880s and early 1900s, see Peck (2000).
